# Development and application of a scoring and visualization approach for 24-hour movement behaviours: an example based on social-emotional development in early years children

**DOI:** 10.1186/s12966-026-01907-y

**Published:** 2026-03-24

**Authors:** Samah Zahran, Dorothea Dumuid, Mark S. Tremblay, Dylan P. Cliff, Devan Antczak, Eivind Aadland, Emad A. Anam, Katrine N. Aadland, Hayley Christian, Jade Burley, Catherine E. Draper, Diego Augusto Santos Silva, Esther M.F. van Sluijs, Timothy S. Olds, Ty Stanford, Rute Santos, Zhiguang Zhang, Ian Janssen

**Affiliations:** 1 School of Kinesiology and Health Studies, Queen’s University, Kingston, Ontario Canada; 2 Alliance for Research in Exercise, Nutrition and Activity (ARENA), Allied Health & Human Performance, University of South Australia, Adelaide, Australia; 3 Healthy Active Living and Obesity Research Group, Children’s Hospital of Eastern Ontario Research Institute, Ottawa, Ontario Canada; 4 Department of Pediatrics, University of Ottawa, Ottawa, Ontario Canada; 5 School of Education, Early Start, Faculty of the Arts, Social Sciences & Humanities, University of Wollongong, Wollongong, Australia; 6 Department of Sport, Food and Natural Sciences, Faculty of Education, Arts and Sports, Western Norway University of Applied Sciences, Sogndal, Norway; 7 Orthopedic Surgery Department, Faculty of Medicine, King Abdulaziz University, Jeddah, Saudi Arabia; 8 School of Population and Global Health and The Kids Research Institute Australia, The University of Western Australia, Perth, Western Australia Australia; 9 SAMRC Developmental Pathways for Health Research Unit, Department of Paediatrics, Faculty of Health Sciences, University of the Witwatersrand, Johannesburg, South Africa; 10 Sports Center, Department of Physical Education, Federal University of Santa Catarina, Florianopolis, Santa Catarina Brazil; 11 MRC Epidemiology Unit, School of Clinical Medicine, University of Cambridge, Cambridge, UK; 12 Institute of Education and Research Centre on Child Studies, University of Minho, Braga, Portugal; 13 Department of Public Health Sciences, Queen’s University, Kingston, Ontario Canada

**Keywords:** Physical activity, Sedentary behaviour, Sleep, Compositional data analysis, Early childhood

## Abstract

**Background:**

Current methods for assessing the healthfulness of 24-hour movement behaviours (sleep, sedentary time, light physical activity, moderate-to-vigorous physical activity) use binary classifications that fail to capture their continuous and compositional nature. This study introduces a percentile-based scoring and visualization approach to evaluate the healthfulness of movement behaviour time-use compositions, using social-emotional development in early childhood as an example.

**Methods:**

This cross-sectional study includes 560 children aged 1.2–2.9 years and 1,500 children aged 3.0-4.9 years from Sleep and Activity Database for the Early Years (SADEY), an international accelerometer repository of young children’s movement behaviours. Sedentary time, light physical activity, and moderate-to-vigorous physical activity were measured using accelerometers. Sleep duration was parent-reported. Social-emotional development was assessed using age- and sex-normalized scores from the Strengths and Difficulties Questionnaire. Linear regression models with compositional covariates were used to model associations between movement behaviours and Strengths and Difficulties Questionnaire scores. Representative grids containing all possible time-use compositions (in 5 min/d increments) of sleep, sedentary time, light physical activity, and moderate-to-vigorous physical activity were developed. The regression models were applied to each time-use composition in the grid, and the predicted scores were ranked to create percentile scores for different movement behaviour time-use compositions.

**Results:**

The 24-hour movement behaviour composition was associated with all five Strengths and Difficulties Questionnaire scores in both age groups (*p* ≤ 0.01). The grids contained 17,577 and 16,535 possible time-use compositions for 1–2 and 3–4-year-olds, respectively. Time-use compositions ranked at the 0th percentile had the least sleep and highest sedentary time, while those ranked at the 100th percentile had the most sleep and least sedentary time. Across the central range of the percentile score distribution (e.g., rankings between the 25th to 75th percentiles), some very different time-use compositions had the same percentile score. Interactive visualization tools were presented to enable real-time exploration of percentile scores for various movement behaviour time-use compositions.

**Conclusions:**

This study introduces a novel approach to evaluate the health benefits of movement behaviours. This approach moves beyond traditional binary cutoffs to recognize the gradual improvements in health that occur with small changes in behaviours, and that there are multiple pathways to achieving the same health benefits.

**Supplementary Information:**

The online version contains supplementary material available at 10.1186/s12966-026-01907-y.

## Background

 Sleep, sedentary behaviour (SED), and physical activity (PA) together make up the 24-hour day [1]. Because time spent in any one of these movement behaviours necessarily displaces time in others, these behaviours are inherently co-dependent and are best examined collectively rather than in isolation [1, 2]. Recognizing this co-dependence, Canada introduced the 24-Hour Movement Guidelines for children and youth aged 5 to 17 years, which were later extended to other ages and countries [3, 4]. These guidelines marked a paradigm shift by moving from a focus on individual movement behaviours to an integrated approach that considers the balance of sleep, SED, and PA [3]. For instance, the guidelines for 3- to 4-year-olds recommend 10–13 h/day of sleep, no more than 1 h/day of screen time, and at least 3 h/day of total physical activity, including at least 1 h of energetic play [4]. The 24-Hour Movement Guidelines for the Early Years operationalizes light physical activity (LPA) as activities performed at 1.5 to 2.9 metabolic equivalents (METs), and energetic play as moderate-to-vigorous physical activity (MVPA), which corresponds to ≥ 3 METs [5]. 

Although the 24-Hour Movement Guidelines conceptually recognize these movement behaviours as interrelated components of a finite 24-h day, their development was largely informed by studies that considered the behaviours separately or applied statistical methods that did not account for their compositional nature [2]. Since the guidelines were released, a growing body of research has employed compositional data analysis (CoDA), a method that can appropriately account for the co-dependent nature of movement behaviours [6, 7]. A recent systematic review of eight studies examining associations between the 24-hour movement behaviour composition and health indicators in children aged 5 and younger found that the composition was associated with measures of mental health, motor skills and development, and physical fitness [7]. Furthermore, reallocating time into MVPA, particularly from LPA, was beneficial for motor development, while reallocating time from LPA into sleep was unfavourably associated mental health.

Despite the advances that CoDA studies have made to the movement behaviour literature, a central challenge lies in converting CoDA evidence into a usable method for evaluating the healthfulness of an individual’s 24-hour movement composition. A common approach in research and surveillance is to assess adherence to the sleep, SED, and PA recommendations contained in the 24-Hour Movement Guidelines [8, 9]. This approach, however, is not grounded in CoDA. Furthermore, guideline adherence is typically operationalized dichotomously as either meeting or not meeting a recommendation [10]. This dichotomous approach oversimplifies the continuous dose-response relationships between movement behaviours and health observed in both traditional regression and CoDA research [7, 11–13], thereby ignoring important nuances that occur above and below the recommendation threshold [10]. For example, a preschooler who accumulates 5 h/day of total PA would likely experience greater health benefits than one who accumulates 3 h/day, yet both are categorized identically as ‘meeting’ the guideline recommendation because they achieve the 3-hour threshold. As a result, critical dose–response information is lost when behaviours are reduced to dichotomous categories.

Another approach used to assess the healthfulness of an individual’s movement behaviours is the Goldilocks day, which identifies a single time use composition that optimally balances time in sleep, SED, and different intensities of PA [14]. In a recent study of children aged 3 to 5 years, St. Laurent et al. reported that the optimal composition for receptive vocabulary consisted of 12:06 (h: min) of sleep, 4:42 of SED, 5:36 of LPA, and 1:42 of MVPA [15]. These findings illustrate the potential of the Goldilocks approach, but also raise questions about feasibility, as the movement behaviour levels required to meet the optimal day may be unrealistic for a large portion of the population. For example, an MVPA target of 1:42 per day may not be attainable for many preschoolers, nor for caregivers trying to support children in reaching this threshold. In addition, the Goldilocks day provides a single “optimal” composition, disregarding the fact that significant health benefits would be achieved by moving in the right direction, even if optimal targets are not reached. Notwithstanding this limitation, the Goldilocks day approach benefits from its grounding in CoDA, which is specifically designed for data representing parts of a whole [2]. 

Taken together, the shortcomings of both dichotomous guideline adherence and the Goldilocks approach underscore the need for methods for determining the healthfulness of movement behaviour compositions that are interpretable and capable of capturing graded health benefits. Precedents for such approaches can be found in other areas of health research, where composite scoring systems have been developed to integrate multiple continuous risk factors into a single index. A widely used example is the Framingham Risk Score in cardiovascular epidemiology, which combines age, sex, cholesterol, blood pressure, smoking, and diabetes status to generate a continuous index of 10-year absolute risk [16]. Despite its complexity, the Framingham Risk Score is widely used in clinical practice to assess risk and guide heart disease prevention efforts [17]. The score can be determined using online calculators and mobile applications, which allows healthcare providers to efficiently assess risk and personalized prevention strategies [17]. 

To address the limitations of both guideline adherence and the Goldilocks approach, new methods are needed that are grounded in CoDA, treat movement behaviours as continuous variables, and provide practical tools for surveillance, clinical application, and personalized feedback. In this paper, we introduce a novel scoring and visualization method that evaluates the healthfulness of 24-hour movement behaviours on a percentile-based continuum. This approach acknowledges that all movement behaviours contribute to health, that the health benefits exist along a continuum, and that different time-use compositions could yield equivalent health outcomes. The specific purpose of our study was to develop and demonstrate the application of a movement behaviour percentile score based on social-emotional indicators obtained in a sample of young children. Although demonstrated here in early childhood, this scoring framework could be extended to other health outcomes and applied across diverse population groups.

## Methods

### Study design and participants

This cross-sectional study is based on the Sleep and Activity Database for the Early Years (SADEY), an ongoing international collaboration that pools accelerometer-measured movement behaviour data [18]. SADEY includes data from 8,409 children aged ≤ 7 years from 14 studies conducted in 7 countries. These studies were based on convenience samples. Ethical approval was obtained for SADEY and all contributing studies. In each study, children wore an Actigraph accelerometer on their waist for a minimum of one day. The accelerometer data were uniformly reprocessed using a standardized approach. Additionally, various health and developmental outcomes, as well as sociodemographic covariates, were harmonized.

Four contributing SADEY studies were included in the present study: PATH-ABC [19], ACTNOW [20], Get-Up! [21], and PLAYCE [22]. Data from these studies were used to investigate the association between the movement behaviour composition (sleep, SED, LPA, and MVPA) and five domains of social-emotional development measured using the Strengths and Difficulties Questionnaire (SDQ) [23]. Analyses were conducted separately for 1 to 2-year-olds and 3 to 4-year-olds. Minimum age was 14 months, and all children were ambulatory at the time of assessment. Boys and girls were combined because sex interaction terms were not significant.

### Measures

#### Movement behaviours

Total sleep duration, including naps, was assessed by a single parental-reported item in PATH-ABC, ACTNOW, and PLAYCE. In Get-Up!, sleep duration was estimated based on parent-reported bedtime and wake-up times. SED, LPA, and MVPA were measured using ActiGraph GT3X+ accelerometers (ActiGraph Corporation, Pensacola, FL) worn on the right hip. Data were processed with the PhysicalActivity package in R (version 4.2.2) following the SADEY protocol [18]. 

Raw accelerometer data were collapsed into 15-s epochs, with non-wear time defined as ≥ 20 consecutive minutes of zero counts. A valid day required ≥ 6 h of wear during waking hours. Participants required ≥ 3 valid days (average: 5 days) for inclusion. A cutoff of ≤ 25 counts/15s distinguish SED from LPA [24] and a cutoff of ≥ 420 counts/15s distinguished MVPA from LPA [25]. These cut-points are more accurate at classifying movement intensity in the early years than other cut-points commonly used in the literature [26]. 

The ActiGraph demonstrates acceptable reliability in early years children when estimating the consistency of accelerometer estimates of PA and SED across days from a single measurement period [27]. When comparing measurement periods over multiple seasons, the ActiGraph shows moderate reliability [28]. 

The average ± SD waking wear times (sum of SED, LPA and MVPA) were 9:01 ± 2:02 and 9:55 ± 2:06 for the 1 to 2-year-olds and 3 to 4-year-olds age groups, respectively. We assumed that non-wear time occurred during waking hours and was distributed proportionally among SED, LPA, and MVPA based on their recorded durations during wear time. Thus, non-wear time was imputed by linearly adjusting time in waking behaviours to sum to 24 h minus sleep duration, so that average total movement behaviour time added up to exactly 24 h/day [29]. The average and range (minimum to maximum) imputed time for 1 to 2-year-olds was 1:47 (1:10 to 6:15) for SED, 1:47 (1:10 to 6:55) for LPA, and 0:24 (0:11 to 2:22) for MVPA. For 3 to 4-year-olds, the average imputed time was 1:29 (1:23 to 6:36) for SED, 1:34 (1:16 to 2:25) for LPA, and 0:22 (0:15 to 3:36) for MVPA.

#### Social-emotional development

Social-emotional development was assessed using the SDQ, a standardized tool for evaluating behavioural issues [30]. The SDQ was originally developed for children aged 2 years and older, though it has also been applied in younger toddlers [23, 31]. The SDQ is a 25-item caregiver-completed questionnaire that measures five domains: hyperactivity, emotional symptoms, conduct problems, peer problems, and prosocial behaviour [23]. For the hyperactivity, emotional symptoms, conduct problems, and peer relationship problems, scores were multiplied by -1 so that higher scores indicate better social-emotional development.

Because SDQ scores differ in boys and girls and improve as a function of normal maturation, sex- and age-normalized values were created for analysis. Each SDQ score was regressed up to a full cubic polynomial in age separately within boys and girls. The standardized residuals represented the sex- and age-normalized z-scores used in the linear regression.

#### Covariates

Parental household status, parental education, and study were controlled for as confounders. These were selected based on their known associations with movement behaviours and socio-emotional development and availability in contributing SADEY studies.

### Data treatment and statistical analysis

Statistical analyses were conducted in R 4.2.2 [32]. Descriptive statistics were used to summarize the variables, including the geometric means the movement behaviours.

#### Developing the scoring system for the 24-hour movement behaviour composition

We used a multi-step approach to create a percentile score that reflected the healthfulness of 24-hour movement behaviours. This approach is a modification to the Goldilocks day CoDA approach [14] and was based on the associations between sleep, SED, LPA, and MVPA with the sex- and age-normalized SDQ z-scores.

Step 1: Linear regression models were used to examine the relationships between movement behaviours and the sex- and age-normalized SDQ z-scores. Separate models were created for each SDQ domain z-score. The independent variables were the confounders and an isometric log-ratio (ilr) coordinate system comprised of sleep, SED, LPA, and MVPA. For each SDQ domain z-score, a series of four models were used, with each model rotating through the ilr transformations so that different behaviours were placed in the numerator. For illustration purposes, the ilr coordinates for MVPA were:

*zi*1 = $\:\sqrt{\frac{3}{4\:}}In\frac{MVPA}{\sqrt[3]{LPA\times\:SED\times\:sleep}}$, *zi*2 = $\:\sqrt{\frac{2}{3\:}}In\frac{LPA}{\sqrt{SED\times\:sleep}}$, and *zi*3 =$\:\sqrt{\frac{1}{2\:}}In\frac{SED}{Sleep}$

Quadratic terms for the isometric log-ratio coordinates were tested using orthogonal polynomial regression to explore non-linear relationships. Quadratic terms were retained if they significantly improved model fit.

 Step 2: A grid of data points in 5-minute increments, that reflect the plausible time-use behaviour compositions in the sample, was created using the ring-fencing approach [33]. This grid represented all possible combinations of the four movement behaviours in 24-hours. The ring-fencing approach calculated Mahalanobis distances from the mean vector and covariance matrix of the ilr-transformed data, reflecting each grid point’s distance from the data center. Thresholds between the 70th and 90th percentiles (in 5-percentile increments) were tested, with the 80th percentile selected as the one that captured the highest density—and most representative—time-use compositions. After applying the fencing, the grid contained 17,577 possible time-use compositions for 1 to 2-year-olds, and 16,535 for 3 to 4-year-olds.

Step 3: The regression models created in Step 1 were used to predict the SDQ z-scores for each 24-hour movement behaviour time-use composition in the grids generated in Step 2.

Step 4: The five predicted SDQ z-scores generated in Step 3 were summed together, resulting in a total predicted SDQ score.

Step 5: Percentile scores were assigned to the total predicted SDQ z-score calculated in Step 4. The time-use composition from the grid with the highest sum was assigned the 100th percentile, while the composition with the lowest sum was assigned the 1st percentile.

The percentile scores created in Step 5 reflect the final continuous score that reflects the healthfulness of any possible 24-hour movement behaviour time-use composition.

#### Presentation and customization of the 24-hour movement behaviour scoring system

It was impractical to present percentile scores for all time-use compositions in a table. Instead, we present in tabular format a few examples of time-use compositions that occurred at the 0st, 10th, 25th, 50th, 75th, 90th, and 100th percentiles. The presentation of percentile scores for all possible time-use compositions was in the form of a colour-coded 3-dimensional isobar in an interactive online interface. In this interface, the user can hover over the isobar to view the percentile score corresponding to any specific time-use composition. Additionally, percentile scores were shown in the paper using a colour-coded ternary diagram. To present the ternary diagram in a 2-dimensional format, LPA and MVPA were merged into total PA, and the 3 axes on the ternary diagram were sleep, SED, and total PA.

We also developed a Shiny app interactive web application using the Shiny package in R. Users can adjust sliders to select a specific time-use composition. After doing so, the percentile score for that time-use composition is shown. Any possible 24-hour movement behaviour time-use composition can be selected as long as two conditions are met: [1] the total of time spent in sleep, SED, LPA, and MVPA must equal exactly 1440 min (24 h) per day, and [2] the selected time-use composition is within the grids of the possible time-use compositions generated in Step 2.

## Results

A total of 2,060 children were included: 560 children aged 1.2 to 2.9 years and 1,500 children aged 3.0 to 4.9 years (Figure S1). Descriptive characteristics of participants are in Table 1. Median SDQ scores, before multiplying by -1 for hyperactivity, emotional symptoms, conduct problems, and peer relationship problems and generating sex- and age-normalized z-scores, were ≤ 4/10 for these domains and ≥ 7/10 for prosocial behaviour.

**Table 1:** Participant characteristics

Variable	1 to 2-year-olds	3 to 4-year-olds
Sex, n (%)		
Boys	303 (54.1)	785 (52.3)
Girls	257 (45.9)	715 (47.7)
Age, mean years (SD)	2.4 (0.5)	3.9 (0.5)
Parental education, n (%)		
< high school	11 (2.5)	30 (2.0)
≥ high school	549 (97.5)	1470 (98.0)
Parental household status, n (%)		
Co-parenting household	516 (92.1)	1353 (90.2)
Single parent	44 (7.9)	147 (9.8)
Social-emotional development scores, median (IQR)		
Emotional symptoms	1 (0-2)	1 (0-2)
Conduct problems	2 (1-3)	1 (0-3)
Hyperactivity	4 (2-5)	3 (2-5)
Peer problems	1 (0-2)	1 (0-2)
Prosocial behaviour	7 (6-8)	8 (6-9)
Movement behaviours*		
Sleep	10:51	10:40
Sedentary time	5:46	5:35
Light physical activity	5:55	6:06
Moderate-to-vigorous physical activity	1:28	1: 39

Social-emotional development scores are based on raw scores

*SD* standard deviation, *IQR* interquartile range

*Presented as compositional center. Calculated as the geometric means of each activity adjusted so that the sum of the four movement behaviours was exactly 24 hr/day

The movement behaviour composition was significantly associated with all five sex- and age-normalized SDQ z-scores in both age groups (*p* ≤ 0.01, see Table 2). Quadratic terms improved model fit and were retained, indicating non-linear associations. Table 2 summarizes the primary direction of association for each movement behaviour based on the predicted effect from the regression models. The direction of the arrows in the table indicates the primary direction of the associations. Overall, relative time spent in sleep was positively associated with most SDQ z-scores, while relative time spent in SED was negatively associated with most SDQ z-scores (Table 2). Associations for LPA and MVPA were mixed (Table 2). The specific β coefficients for the linear and quadratic terms are in Table S1.

**Table 2:** Associations between movement behaviour compositions and sex- and age-normalized SDQ z-scores

Age group and SDQ domain	Model *P* value	Adjusted *R*^2^ for ilrs	Sleep	SED	LPA	MVPA
1 to 2-year-olds						
Emotional symptoms	< 0.001	0.151	↑	↓	↑	↓
Conduct problems	< 0.001	0.122	↑	↓	↔	↓
Hyperactivity	< 0.001	0.083	↑	↓	↔	↓
Peer problems	< 0.001	0.125	↑	↓	↑	↓
Prosocial behaviour	< 0.001	0.107	↓	↑	↓	↑
3 to 4-year-olds						
Emotional symptoms	< 0.001	0.137	↑	↓	↔	↔
Conduct problems	< 0.001	0.132	↑	↓	↔	**↑↓**
Hyperactivity	< 0.001	0.092	↑	↓	↔	**↑↓**
Peer problems	< 0.001	0.135	↑	↓	↔	↔
Prosocial behaviour	< 0.001	0.086	↓	↑	↓	↔

The arrows indicate the primary direction of association between each movement behaviour (relative to the remaining behaviours) and SDQ outcomes. Non-linearity was accounted for by including orthogonal polynomial terms (linear and quadratic) in the regression models. All estimates were adjusted for parental education, parental household status, and study.**↑** positive association**↓** negative association**↑↓** positive association at low MVPA levels and negative association at high MVPA levels**↔** non-significant association (*p* ≥ 0.05).

*SDQ* Strengths and difficulties questionnaire *ilr* isometric log-ratio coordinate, *SED* sedentary time *LPA* light physical activity, *MVPA* moderate-to-vigorous physical activity

The grid of 17,577 possible time-use compositions in 1 to 2-year-olds and 16,535 possible time-use compositions in 3 to 4-year-olds was developed. The regression models capturing the associations between movement behaviours and SDQ z-scores were applied to each time-use composition in the grids, and the predicted scores that resulted were ranked to create the movement behaviour percentile scores. Table 3 presents examples of time-use compositions for hypothetical children with specific movement behaviour percentile scores. Overall, a hypothetical child at the 0th percentile (worst) had a time-use composition containing less sleep and more SED than the other examples. A hypothetical child at the 100th percentile (best) had a time-use composition containing more sleep and less SED than the other examples. Across the central range of the percentile score distribution (e.g., 25th, 50th, 75th percentiles), hypothetical children with the same percentile score had very different time-use compositions.

**Table 3:** Time-use compositions for hypothetic children with specific movement behaviour percentile scores

Example No.	Movement Behaviour Percentile Score	Time-use composition (hours:minutes)			
		Sleep	SED	LPA	MVPA
1 to 2-year-olds					
1	0th	9:05	7:05	6:40	1:10
2	10th	8:50	6:55	6:35	1:40
3	25th	11:30	5:50	5:00	1:40
4	25th	9:35	6:20	6:15	1:50
5	50th	10:50	5:50	5:40	1:40
6	50th	10:05	5:35	6:30	1:50
7	50th	12:00	4:55	5:10	1:55
8	75th	11:55	5:00	3:45	1:40
9	75th	12:15	4:45	5:15	1:45
10	90th	12:45	5:35	4:45	0:55
11	100th	13:00	4:35	5:20	1:05
3 to 4-year-olds					
1	0th	8:55	7:25	6:20	1:20
2	10th	10:40	7:05	5:05	1:10
3	25th	10:40	6:35	5:05	1:40
4	25th	10:20	6:25	5:30	1:45
5	50th	11:40	6:20	4:50	1:10
6	50th	11:10	6:05	5:35	1:10
7	50th	10:30	5:40	6:25	1:25
8	75th	11:55	5:30	4:55	1:40
9	75th	10:35	4:35	7:00	1:50
10	90th	12:20	5:00	5:00	1:40
11	100th	12:45	3:45	5:20	1:40

*SED* sedentary behaviour, *LPA* light physical activity, *MVPA* moderate-to-vigorousphysical activity. The 0^th^ percentile indicates the lowest (worst) score and the 100^th^ percentile indicates the highest (best) score

**Fig. 1:**
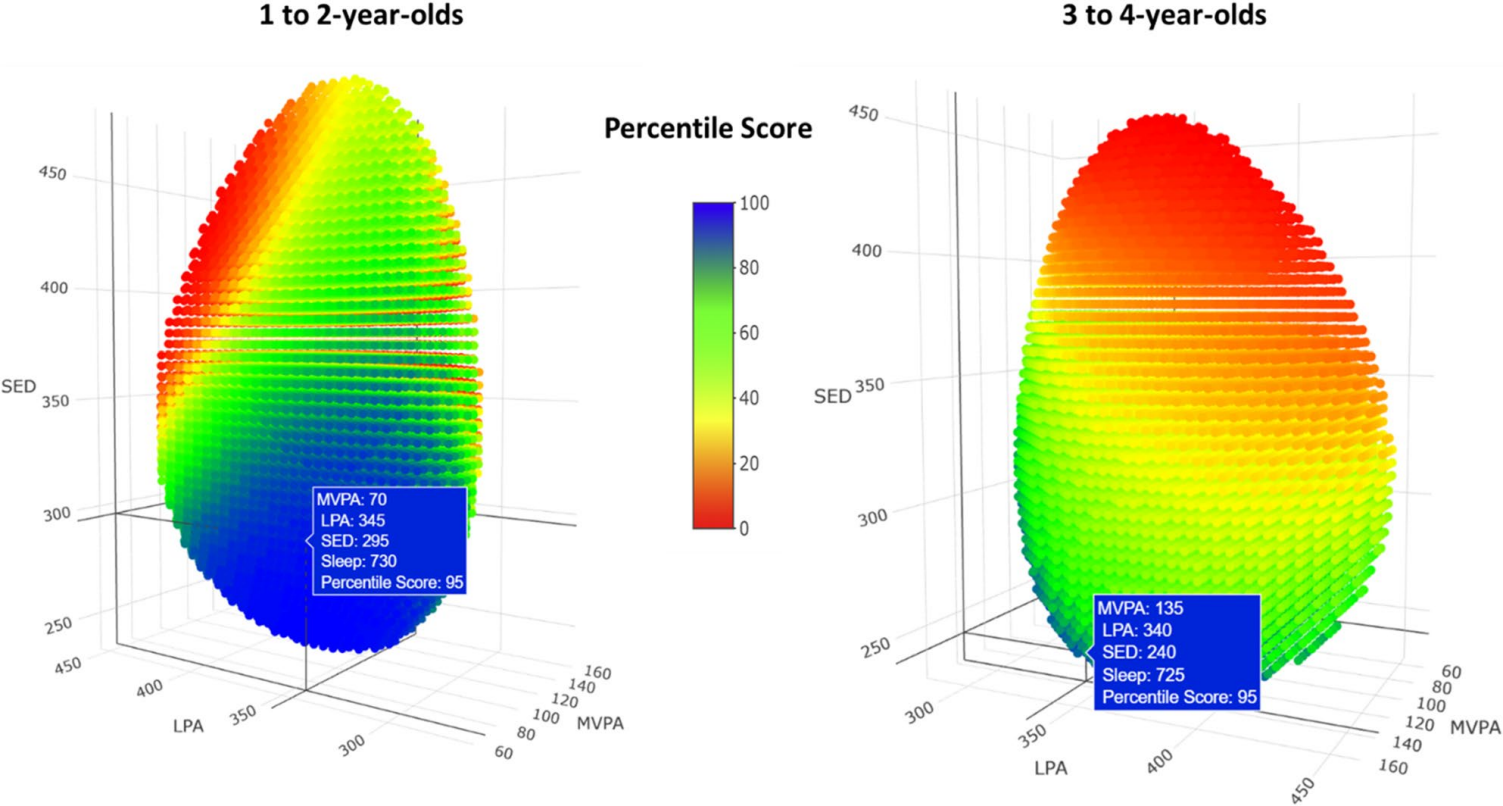
Two-dimensional illustration of the isobar of movement behaviour percentile scores for all time-use compositions. SED, sedentary behaviour; LPA, light physical activity; MVPA, moderate-to-vigorous physical activity. The 1st percentile indicates the lowest (worst) score, and the 100th percentile the highest (best) score. Links for the three-dimensional isobar apps for 1 to 2-year-olds (Available from: https://samahzahran.github.io/new_isobar_young1/3d_plotisobar_youngnew.html) and 3 to 4-year-olds (Available from: https://samahzahran.github.io/new_isobar_old/3d_plotisobar_oldnew.html)

**Fig. 2:**
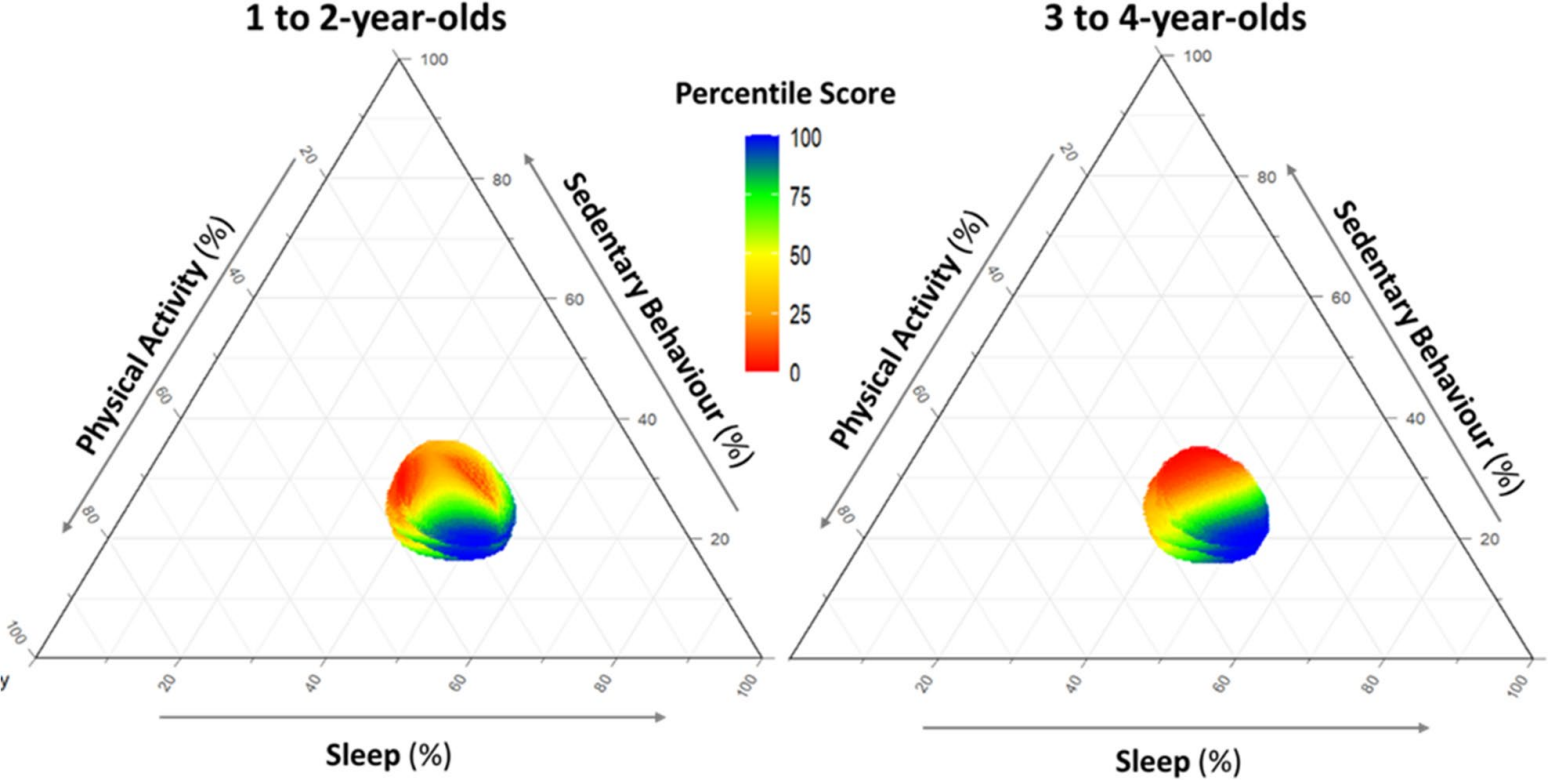
Ternary diagram of the movement behaviour percentile score for all time-use compositions based on sleep, sedentary behaviour, and total physical activity (light + moderate-to-vigorous). The 1st percentile indicates the lowest (worst) score, and the 100th percentile the highest (best) score

The isobars for 1 to 2-year-olds (Available from: https://samahzahran.github.io/new_isobar_young1/3d_plotisobar_youngnew.html) and 3 to 4-year-olds (Available from: https://samahzahran.github.io/new_isobar_old/3d_plotisobar_oldnew.html) provide an interactive online interface that allows users to visualize how movement behaviour percentile scores vary across the grid of time-use compositions. Users can rotate the axes of the isobar and hover over specific time-use combinations to see their corresponding percentile scores. A 2-dimensional image of one plane of each isobar is provided in Fig. 1. The ternary plots in Fig. 2 further illustrate how SDQ z-scores differ across time-use compositions.

**Fig. 3:**
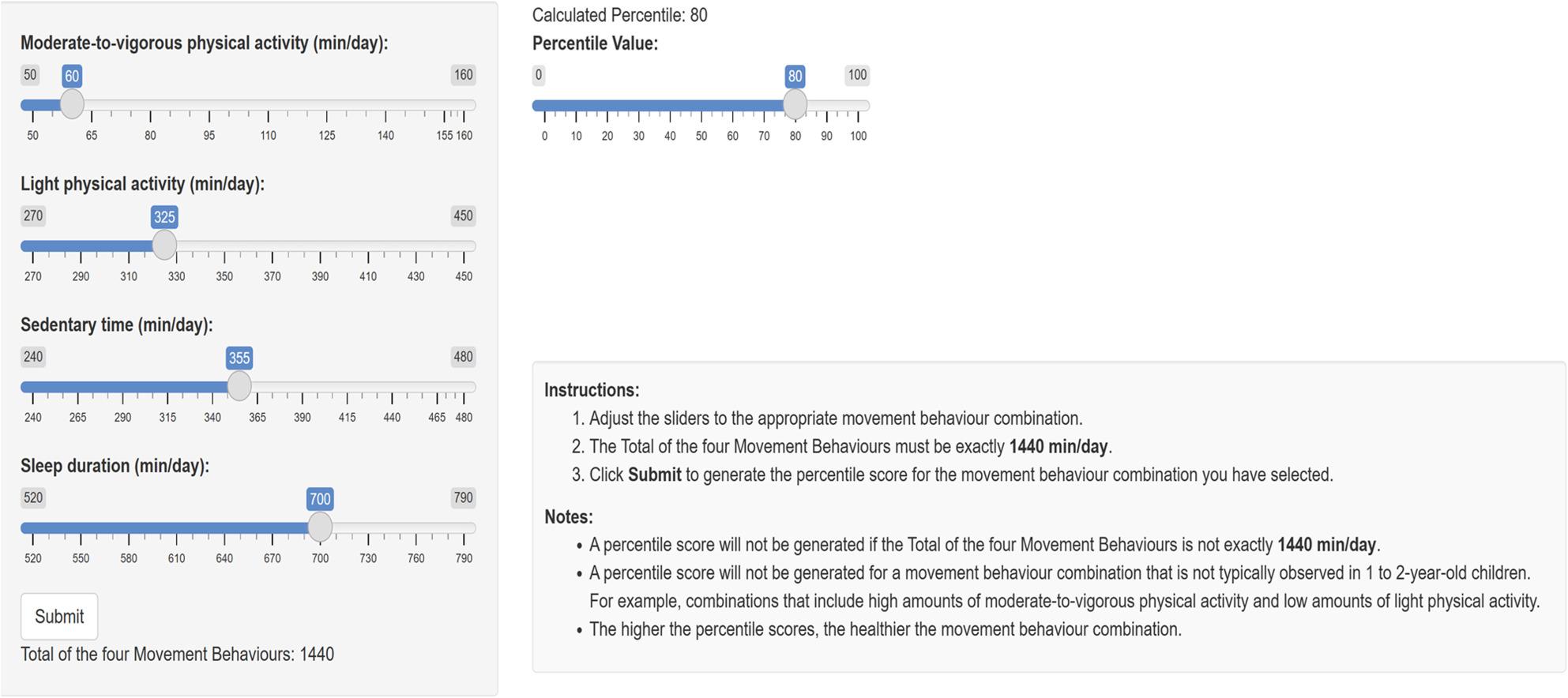
Illustration of the interactive tool that can be used to determine movement behaviour percentile scores for specific time-use compositions. When using the tool, the user adjusts the sliders for each movement behaviour to a desired time-use composition that sums to exactly 1440 min (24 h) per day. Links for the interactive tool for 1 to 2-year-olds (Available from: https://samah.shinyapps.io/Children1-2/) and 3 to 4-year-olds (Available from: https://samah.shinyapps.io/children3-4/)

An app was developed to provide users with an interactive tool to determine movement behaviour percentile scores for specific time-use compositions. The user adjusts the sliders for each movement behaviour to a desired time-use composition adding up to exactly 1440 min (24 h) per day. A picture of the app interface is in Fig. 3. The app can be accessed at the following links for 1 to 2-year-olds (Available from: https://samah.shinyapps.io/Children1-2/) and 3 to 4-year-olds (Available from: https://samah.shinyapps.io/children3-4/).

R codes for the main analysis, including the codes used to develop the two interactive apps, are provided in a supplementary file 2.

## Discussion

This paper introduces a novel scoring and visualization method that evaluates the health benefits of 24-hour movement behaviors on a percentile-based continuum. Developed using social-emotional indicators in early childhood, this method has the potential for broader application to various health outcomes across diverse populations. In the discussion section, we highlight the method’s strengths, explore challenges with its application, and suggest future directions.

A major strength of our newly developed approach is its ability to capture the continuous nature of the associations between 24-hour movement behaviours and health outcomes. Traditional assessments that rely on guideline recommendations use binary classifications (e.g., meeting versus not meeting recommendations) or ordinal cutoffs [10], which overlook the compositional nature of movement behaviours and their dose-response relationships with health outcomes. The concept of integrating multiple variables into a continuous risk score is well established in other fields, such as the Framingham Risk Score for cardiovascular disease that incorporates multiple risk factors to assess risk and guide heart disease prevention efforts [16, 17]. Similarly, our percentile-based scoring system should be paired with the accompanying interactive digital tools to facilitate its use. These tools could help users input movement behaviour data, receive personalized feedback, and explore ways to optimize their child’s movement health [10]. 

Another advantage of our continuous score approach versus a binary classification is its capacity to track the health impacts of incremental changes in movement behaviour. This method acknowledges that small improvements in movement behaviours can yield meaningful health benefits, even if an individual does not surpass a recommended threshold. From a behaviour change perspective, a tracking system that provides graded feedback and allows for progressive adjustments may foster sustained engagement and long-term health improvements [10]. The importance of considering the continuous nature of movement behaviours was also apparent for children who are already on the healthy side of the 24-Hour Movement Guideline cutoffs. For example, the movement behaviour compositions at the 90th to 100th percentiles for the 3- to 4-year-olds in our study were characterized by having a sleep duration near the upper end of the healthy sleep range, SED levels that were amongst the lowest in the sample, and PA levels that greatly exceeded the guideline targets (i.e., ~ 5 h of LPA and close to 2 h of MVPA).

Another key strength of the percentile-based scoring approach is that it captures the compositional nature of movement behaviours by acknowledging that the health benefits of any one behaviour depend on the levels of the others. For example, two children with the same MVPA level may experience different health benefits depending on how their remaining time is allocated across sleep, LPA, and SED. By comparison, the 24-Hour Movement Guidelines prescribe fixed recommendations for each movement behaviour. Furthermore, our findings demonstrate that individuals with extremely different time-use compositions can achieve the same percentile score, reinforcing that multiple movement patterns can lead to comparable health benefits. This offers flexibility by allowing individuals to improve their score in ways that align with their personal circumstances and preferences.

Guideline-adherence studies consistently show that toddlers and preschoolers who meet more of the 24-h guidelines recommendations demonstrate better executive function, psychosocial outcomes, and social-cognitive development than those who meet only one, two, or none of the recommendations [8, 9, 34]. CoDA studies of early years children have routinely found that the 24-hour time-use composition of movement behaviours is associated with health and developmental indicators [7]. Similarly, we found that to achieve the highest percentile scores, children needed to do well across multiple movement behaviours simultaneously. Together, these complementary results suggest that benefits accumulate when multiple behaviours move in a favourable direction together.

A major challenge of implementing a scoring system such as the one developed here is its practical utility for messaging. Existing 24-hour Movement Guidelines contain simple, prescriptive recommendations, making them easier to communicate and understand. One possible suggestion is to use precision health tools such as interactive apps, artificial intelligence (AI)-driven feedback systems, and wearable technology, which make it possible to create personalized movement behaviour recommendations, ensuring that complex data is translated into actionable insights that are clear and personalized [10]. Another challenge is fitting a continuous percentile-based scoring approach within public health surveillance systems that typically track prevalence-based metrics (e.g., prevalence meeting specific guideline recommendations) [35]. Because our method produces a continuous score, a different approach would be required. A potential solution is to establish percentile scores for each time-use composition based on a reference population and use this standard to assess other samples across different populations and time points. This approach would mirror how body mass index growth references are used to calculate body mass index percentiles and determine and compare the prevalence of childhood obesity [36]. 

We have a few suggestions on how our percentile-scoring system could be refined in future studies. First, this approach should be explored in other populations with other health and development outcomes. Second, the scoring approach could be customized to prioritize specific health outcomes by assigning differential weightings to each outcome based on its relative importance. Third, the SED component of the scoring system could be further divided into screen-based and non-screen sedentary activities, which would provide a more accurate assessment of the differential effects of sedentary time on health. Fourth, the PA components of the scoring system could be further customized to account for activity patterns (e.g., prolonged bouts vs. breaks) and intensities (e.g., moderate vs. vigorous), particularly when applied to adults. Finally, integrating precision health tools such as AI-driven modeling, machine learning algorithms, and digital tracking applications could further enhance individualized movement recommendations [10]. It is possible that percentile scores could be specific to a person’s age, gender, race, etc.

There are limitations specific to the study sample rather than the scoring system. The accelerometer cut-points applied were validated primarily in 3 to 4-year-olds. When used with toddlers, these thresholds may overestimate LPA and underestimate SED. However, prior work using the same SADEY dataset with similar outcomes, including extensive sensitivity analyses across alternative cut-points, wear-time criteria, and valid-day definitions, showed that findings were consistent [37]. Another noteworthy limitation is that sleep duration was derived from parent-reported measures rather than device-based estimates, which may introduce measurement bias. Finally, the SADEY harmonization process limited the number of demographic variables that could be included in our analyses. While information on age, sex, and select socioeconomic indicators was available, other characteristics (e.g., race/ethnicity) were not uniformly collected and could not be controlled for.

## Conclusions

The percentile scoring system introduced in this study offers a flexible and personalized approach to evaluating 24-hour movement behaviours. By recognizing that health benefits can be achieved through various movement combinations, this system moves beyond the limitations of traditional guidelines and supports the growing emphasis on precision health. Before this scoring system can be implemented in clinical settings, future research should modify the system to include a broader array of health outcomes, evaluate its calibration and discrimination, and confirm external validation [38]. 

## Supplementary Information

Supplementary Material 1.

Supplementary Material 2.

Supplementary Material 3.

## Data Availability

Data were obtained from the Sleep and Activity Database for the Early Years (SADEY), which is a consortium of pooled accelerometer-measured movement behaviour data in children aged 7 years or younger. The data are available by application.

## Abbreviations

SED: Sedentary behaviour
PA: Physical activity
LPA: Light physical activity
MVPA: Moderate-to-vigorous physical activity
METs: metabolic equivalents
CoDA: Compositional data analysis
SADEY: Sleep and Activity Database for the Early Years
SDQ: Strengths and Difficulties Questionnaire
AI: artificial intelligence
