# Supplementary material for: Development and application of a scoring and visualization approach for 24-hour movement behaviours: an example based on social-emotional development in early years children
Source: Int J Behav Nutr Phys Act. 2026 Mar 24;23:46. doi: 10.1186/s12966-026-01907-y (PMC13137636; doi:10.1186/s12966-026-01907-y)
Supplement: Supplementary file 1 — Supplementary Material 1. [file 12966_2026_1907_MOESM1_ESM.docx]

**Supplementary file 1: Supplemental Tables and Figures**

**Figure S1.** Participant flowchart


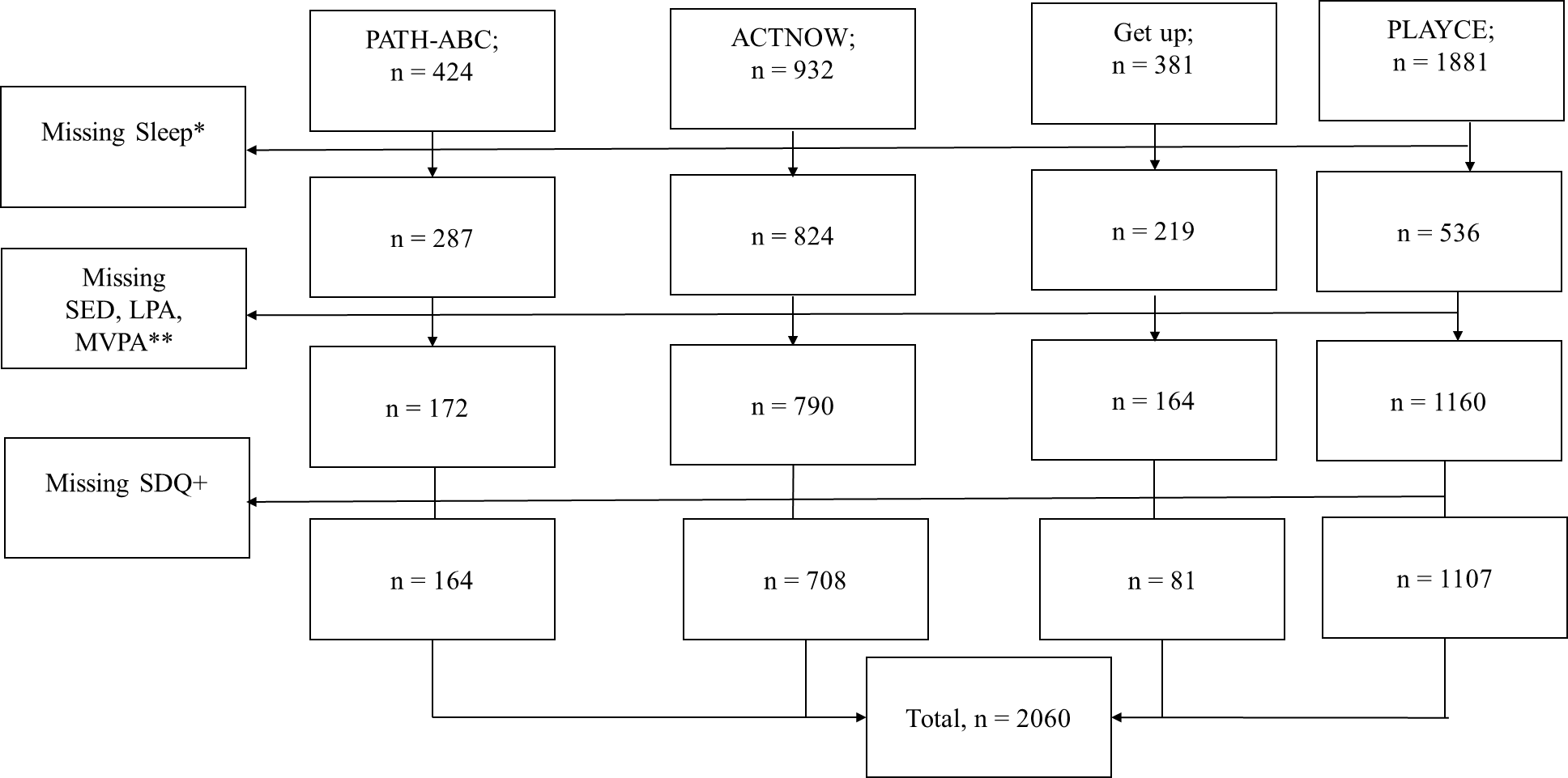


**Figure legend:** Flow chart illustrating exclusion of participants with missing data. *Sleep duration not reported. ** Insufficient accelerometer data to calculate average daily sedentary time (SED), light physical activity (LPA), and moderate-to-vigorous physical activity (MVPA). + Missing data for one or more of the Strengths and Difficulties Questionnaire (SDQ) items.

| **Table S1.** Linear and quadratic coefficients for the association between movement behaviour compositions and sex- and age-normalized SDQ z-scores. | | | | | | | | |
| --- | --- | --- | --- | --- | --- | --- | --- | --- |
| Age group and SDQ domain | β (SE) for | | | | | | | |
|  | Sleep ilr | Sleep ilr² | SED ilr | SED ilr² | LPA ilr | LPA ilr² | MVPA ilr | MVPA ilr² |
| **1 to 2-year-olds** |  |  |  |  |  |  |  |  |
| Emotional symptoms | 5.17 (0.98) * | 1.46 (0.93) | -9.17 (1.13) * | 1.37 (0.90) | 3.67 (0.90) * | -1.60 (0.92) | -4.78 (1.08) * | -1.42 (0.90) |
| Conduct problems | 6.44 (1.00) * | 0.83 (0.95) | -8.30 (1.15) * | 0.19 (0.92) | 1.90 (0.92) | -1.49 (0.93) | -4.06 (1.10) * | -0.95 (0.92) |
| Hyperactivity | 5.25 (1.02) * | 0.81 (0.97) | -6.04 (1.18) * | 1.12 (0.94) | 1.08 (0.94) | -0.77 (0.95) | -3.09 (1.13) * | -0.73 (0.94) |
| Peer problems | 5.05 (0.99) * | 0.88 (0.95) | -8.55 (1.15) * | 0.49 (0.92) | 2.80 (0.92) * | -1.90 (0.93) * | -3.48 (1.10) * | -1.91 (0.92) * |
| Prosocial behaviour | -5.24 (0.99) * | -1.69 (0.95) | 6.97 (1.15) * | -1.57 (0.92) | -1.85 (0.92) * | 0.72 (0.94) | 3.49 (1.11) * | 1.15 (0.92) |
| **3 to 4-year-olds** |  |  |  |  |  |  |  |  |
| Emotional symptoms | 9.74 (0.97) * | 3.30 (0.95) * | -15.71 (1.19) * | -0.89 (0.93) | 1.73 (0.93) | 0.46 (0.95) | -0.56 (1.10) | -1.53 (0.93) |
| Conduct problems | 10.91 (0.97) * | 2.89 (0.95) * | -15.14 (1.18) * | -1.52 (0.93) | 1.25 (0.93) | 0.32 (0.95) | -2.21 (1.10) * | -2.41 (0.93) * |
| Hyperactivity | 9.36 (0.99) * | 2.35 (0.98) * | -11.79 (1.21) * | -3.32 (0.95) * | 0.63 (0.96) | 0.43 (0.98) | -2.42 (1.12) * | -3.19 (0.95) * |
| Peer problems | 11.26 (0.95) * | 3.53 (0.94) * | -16.40 (1.17) * | -1.08 (0.92) | 1.26 (0.92) | 0.36 (0.94) | -1.20 (1.08) | -0.98 (0.92) |
| Prosocial behaviour | -6.70 (1.00) * | -2.02 (0.98) * | 13.09 (1.22) * | 0.49 (0.96) | -2.93 (0.96) * | -0.28 (0.98) | 1.46 (1.13) | 1.96 (0.96) |
| SDQ, Strengths and difficulties questionnaire; ilr, isometric log-ratio; SED, sedentary time; LPA, light physical activity; MVPA, moderate-to-vigorous  physical activity; SE, standard error.  All SDQ domain scores were based on residuals determined by regression a full cubic polynomial for age against the raw SDQ score separately in boys and girls. For all SDQ domains other than prosocial behaviour, the sex- and age-normalized scores were multiplied by -1 so that higher scores indicate better outcomes. All β estimates are ilr regression coefficients that capture the relationships of one behaviour relative to the remaining behaviours. All estimates were adjusted for parental education, parental household status, and study.  * P<0.05. | | | | | | | | |
