# Supplementary material for: Development and application of a scoring and visualization approach for 24-hour movement behaviours: an example based on social-emotional development in early years children
Source: Int J Behav Nutr Phys Act. 2026 Mar 24;23:46. doi: 10.1186/s12966-026-01907-y (PMC13137636; doi:10.1186/s12966-026-01907-y)
Supplement: Supplementary file 2 — Supplementary Material 2. [file 12966_2026_1907_MOESM2_ESM.docx]

# Supplementary file 2: R code

# Development and application of a scoring and visualization approach for 24-hour movement behaviours: an example based on social-emotional development in early years children

Table of Contents

[Preparation for Main Analyses 3](#_Toc194885715)

[Load required libraries that will be used in the analyses 3](#_Toc194885716)

[Download data. 3](#_Toc194885717)

[Close the movement behaviours to 1440 min/day. 3](#_Toc194885718)

[Step 1: Linear regression analysis for SDQ z-scores 4](#_Toc194885719)

[Step 2: Generation of grid of data points in 5-minute increments using the “ring fencing approach” created by Nikfarjam et al (1). 4](#_Toc194885720)

[Step 3: Predicting SDQ z-scores for the grid 9](#_Toc194885721)

[Step 4. Sum the five predicted SDQ z-scores generated in Step 3 to generate a total predicted SDQ score. 10](#_Toc194885722)

[Step 5: Assign percentile scores 11](#_Toc194885723)

[Visualization of the percentile scores 12](#_Toc194885724)

[2-dimensional ternary diagram 12](#_Toc194885725)

[3-dimensional isobar 13](#_Toc194885726)

[Shiny_app 14](#_Toc194885727)

[Ring-Fencing Approach vs. 3 Standard Deviation in Generating Predictive Grids 18](#_Toc194885728)

#

# Preparation for Main Analyses

## Load required libraries that will be used in the analyses

**library**(simplexity)

**library**(compositions)

**library**(codaredistlm)

**library**(knitr)

**library**(purrr)

**library**(ggplot2)

**library**(ggtern)

**library**(gtools)

**library**(Hmisc)

**library**(tidyverse)

## Download data.

Prepare outcome variables (SDQ z-scores) and confounders (parental education, parental marital status, study).

## Close the movement behaviours to 1440 min/day.

Apply Isometric Log-Ratio (ILR) transformation to sleep, sedentary behaviour (SB), light physical activity (LPA), and moderate-to-vigorous physical activity (MVPA).

data$comp = data %>% dplyr::select(sleep,SB,LPA,MVPA)

missingSummary(data$comp) # Ensures data is clean before applying ILR

head(data$comp)

data$ilrs = ilr(data$comp)

data$ilr1 = data$ilrs[,1]

data$ilr2 = data$ilrs[,2]

data$ilr3 = data$ilrs[,3]

head(data)

# Step 1: Linear regression analysis for SDQ z-scores

Linear regression model for the emotional problems SDQ z-score (Emotional_problems).

mod.Emotional_problems = lm(scale((Emotional_problems)) ~ poly(cbind(ilr1, ilr2, ilr3),2) +Maritalstatus+Education+study, data=data)

summary(mod.Emotional_problems)

hist(residuals(mod.Emotional_problems))

car::Anova(mod.Emotional_problems, test.statistic= "F")

res <- resid(mod.Emotional_problems)

plot(fitted(mod.Emotional_problems), res)

abline(0,0)

qqnorm(res)

qqline(res)

plot(density(res))

Repeat this step for the other four SDQ z-scores (conduct problems, hyperactivity, peer problem, and prosocial).

# Step 2: Generation of grid of data points in 5-minute increments using the “ring fencing approach” created by Nikfarjam et al (1).

# ---- grid_sizes ----

clos_val <- 60 * 24 # 1440 minutes

# this is how many points are in D-simplex with m-points per compositional part

n_points <- function(D, m) prod((D + m - 1):(m + 1)) / factorial(D - 1)

# n_points_niave <-

# function(D, m) factorial(D + m - 1) / factorial(m) / factorial(D - 1)

# eg for 4 compositional parts and 1-minutes spacings (i.e., 1440 points)

n_points(4, 1440)

# a table of number of points for different spacings

expand_grid(D = 4, kappa = clos_val, step_size_minutes = c(1, 2, 5, 10, 15, 20, 30)) %>%

mutate(points_per_axis = kappa / step_size_minutes) %>%

mutate(total_grid_pts = map2_dbl(.x = D, .y = points_per_axis, .f = n_points)) %>%

kable(.)

# ---- mean_and_var ----

# normalised one-vs-rest squential binary partition

#V <- ilrBase(D = D, method = "basic")[D:1, (D - 1):1]

#dimnames(V) <- list(c("sleep", "SB", "LPA", "MVPA"), paste0("ilr", 1:3))

#V

# Example D value (replace with actual value if needed)

D <- 4 # Number of components: "sleep", "SB", "LPA", "MVPA"

# Ensure D is correctly defined and is a numeric value

if (!is.numeric(D) || D <= 1) {

stop("D must be a numeric value greater than 1.")

}

# Apply ilrBase to generate the ILR basis matrix

V_base <- ilrBase(D = D, method = "basic")

# Check the dimensions of the resulting matrix

cat("Dimensions of V_base:", dim(V_base), "\n")

# Ensure V_base has the correct number of rows and columns

# The number of rows should be D, and columns should be D-1

if (nrow(V_base) != D || ncol(V_base) != D - 1) {

stop("The dimensions of the ILR basis matrix do not match the expected values.")

}

# Reverse the rows and columns (as per your indexing)

V <- V_base[D:1, (D - 1):1]

# Assign meaningful row and column names

dimnames(V) <- list(c("sleep", "SB", "LPA", "MVPA"), paste0("ilr", 1:3))

# View the resulting matrix

V

# load some real data

#data("fairclough", package = "codaredistlm")

data_example <- data %>% dplyr::select(sleep, SB, LPA, MVPA)

head(data_example)

ilr_dat <- ilr(data_example, V = V)

head(ilr_dat)

ilr_dat <- as.matrix(as.data.frame(ilr_dat))

str(ilr_dat)

(m_ilr <- colMeans(ilr_dat))

(v_ilr <- var(ilr_dat))

# see: ?stats::mahalanobis

lhs_inequality_value <- function(dat, mean_vec = m_ilr, covar_mat = v_ilr) {

mahalanobis(x = dat, center = mean_vec, cov = covar_mat)

}

rhs_inequality_value <- function(percentage = 80, dof = D - 1) {

qchisq(percentage / 100, df = dof)## chi-square critical value threshold

}

ilr_df <-

as_tibble(data_example) %>%

bind_cols(., ilr_dat) %>%

mutate(

lhs_ineq = lhs_inequality_value(ilr_dat),

percentile_from_mean = 100 * pchisq(lhs_ineq, df = 3)

)

head(ilr_df)

percentiles <- seq(5, 95, by = 5)

(obs_perc <- sapply(percentiles, function(x) 100 * mean(ilr_df$percentile_from_mean <= x)))

plot(

0:100, 0:100,

type = "n", bty = "n",

xlab = "Theoretical CDF percentiles",

ylab = "Actual CDF percentiles"

)

points(percentiles, obs_perc, type = "p", pch = 16, col = "magenta")

abline(a = 0, b = 1)

####plot all the percintile ilr data

simplexity::plot_four_comp(

ilr_df,

"sleep", "SB", "LPA", "MVPA",

col = "percentile_from_mean", alpha = 0.5

)

# ---- grid_4_dim_simplex ----

# dim of simplex

D <- 4

# closure value (i.e., total coordinate sum)

# increments of the grid

step_size <- 5 # minutes

# if we want 10 min increments for a 1440 min day

# ===> number of grid points per axis/compositional part = 1440 / 10

(m <- clos_val / step_size)

(npoints_4dim <-

n_points(

D = D, # dim of simplex

m # n_steps across [0, clos_val]

))

d4_grid <- step_size * simplexity::enumerate_simplex(D, m)

colnames(d4_grid) <- rownames(V)

head(d4_grid)

# remove any 0 values

rows_with_zeros <- rowSums(d4_grid == 0) > 0

table(rows_with_zeros, useNA = "ifany")

head(d4_grid[rows_with_zeros, ])

d4_grid <- d4_grid[!rows_with_zeros, ]

head(d4_grid)

# ---- grid_ilrs ----

(grid_n_pts <- nrow(d4_grid))

ilr_grid <- ilr(d4_grid, V = V)

head(ilr_grid)

ilr_grid_df <-

as_tibble(d4_grid) %>%

bind_cols(., ilr_grid) %>%

mutate(

lhs_ineq = lhs_inequality_value(ilr_grid),

percentile_from_mean = 100 * pchisq(lhs_ineq, df = 3),

within_80th_perc = percentile_from_mean <= 80

)

head(ilr_grid_df)

# grid points that remain within fencing

ilr_grid_df %>%

group_by(within_80th_perc) %>%

summarise(

n = n(),

percent_of_grid_pts = 100 * n / grid_n_pts

) %>%

kable(.)

# plot the grid points within fence -- should be similar to fairclough cloud of points

ilr_grid_df %>%

dplyr::filter(within_80th_perc) %>%

simplexity::plot_four_comp(

.,

"sleep", "SB", "LPA", "MVPA",

col = NULL, alpha = 0.25

)

# Filter the dataset based on the within_80_perc variable and select specific columns

# *Thresholds for the fence were tested at intervals between the 70th and 90th percentiles in 5-percentile increments. We applied the same code each time, changing only the percentile value in the following two lines*:

rhs_inequality_value <- function(percentage = 70, dof = D - 1) {

qchisq(percentage / 100, df = dof)## chi-square critical value threshold

}

ilr_grid_df <-

as_tibble(d4_grid) %>%

bind_cols(., ilr_grid) %>%

mutate(

lhs_ineq = lhs_inequality_value(ilr_grid),

percentile_from_mean = 100 * pchisq(lhs_ineq, df = 3),

within_70th_perc = percentile_from_mean <= 70

)

# Step 3: Predicting SDQ z-scores for the grid

Use the results of the regression model generated in Step 1 to predict the emotional problems SDQ z-score for each time-use composition in the grid generated in Step 2.

#1- Emotional problem z-score:

pred <- predict(

mod.Emotional_problems,

newdata = data.frame(

ilr1 = ilr.grid[, 1],

ilr2 = ilr.grid[, 2],

ilr3 = ilr.grid[, 3],

Maritalstatus = rep(mean(data$Maritalstatus), nrow(griddata)),

study=rep(mean(data$ study),nrow(griddata)),

Education = rep(mean(data$Education), nrow(griddata))

),

re.form = NA

)

#this makes a dataframe with the predictive grid and the outcome score

df.Emotional_problems=data.frame(sleep=griddata[,1],Sedentary=griddata[,2], LPA=griddata[,3], MVPA=griddata[,4], Predicted_Outcome=pred)

#put activity variables in minutes/day

df.Emotional_problems$sleep=df.Emotional_problems$sleep*1440

df.Emotional_problems$Sedentary=df.Emotional_problems$Sedentary*1440

df.Emotional_problems$LPA=df.Emotional_problems$LPA*1440

df.Emotional_problems$MVPA=df.Emotional_problems$MVPA*1440

Repeat this step for the other four SDQ z-scores (conduct problems, hyperactivity, peer problem, and prosocial).

# Step 4. Sum the five predicted SDQ z-scores generated in Step 3 to generate a total predicted SDQ score.

#First change the columns name to sum them

colnames(df.Emotional_problems)[colnames(df.Emotional_problems) == "Predicted_Outcome"] <- "Predicted_Outcome_emotional"

colnames(df.Conduct_Problems)[colnames(df.Conduct_Problems) == "Predicted_Outcome"] <- "Predicted_Outcome_conduct"

colnames(df.Hyperactivity)[colnames(df.Hyperactivity) == "Predicted_Outcome"] <- "Predicted_Outcome_hyperactivity"

colnames(df.Prosocial)[colnames(df.Prosocial) == "Predicted_Outcome"] <- "Predicted_Outcome_prosocial"

colnames(df.Peer_Problems)[colnames(df.Peer_Problems) == "Predicted_Outcome"] <- "Predicted_Outcome_peer"

# List of data frames to combine

df_list <- list(df.Peer_Problems, df.Emotional_problems, df.Conduct_Problems, df.Prosocial, df.Hyperactivity)

# Define the common columns

common_cols <- c("MVPA", "LPA", "Sedentary", "sleep")

# Use reduces to iteratively merge the data frames

combined_df <- reduce(df_list, function(x, y) merge(x, y, by = common_cols, all = TRUE))

# Create new column Predicted_Outcome_total by summing individual columns (z-scores)

combined_df$Predicted_Outcome_total <-

rowSums(combined_df[c("Predicted_Outcome_peer", "Predicted_Outcome_emotional",

"Predicted_Outcome_conduct", "Predicted_Outcome_prosocial",

"Predicted_Outcome_hyperactivity")], na.rm = TRUE)

# Step 5: Assign percentile scores

Assign percentile scores to each time-use composition generated in the grid.

# Calculate percentiles for total predicted outcomes z-scores

combined_df$percentile_total <- ecdf(combined_df$Predicted_Outcome_total)(combined_df$Predicted_Outcome_total) * 100

combined_df$percentile_total1 <- round(ecdf(combined_df$Predicted_Outcome_total)(combined_df$Predicted_Outcome_total) * 100)

# Visualization of the percentile scores

## 2-dimensional ternary diagram

Ternary diagrams are limited to three variables. Sum light physical activity and moderate-to-vigorous physical activity to create a total physical activity variable. Include total physical activity, sleep, and sedentary behaviour in the ternary diagram.

# First add a new variable PA to the dataset

combined_df$PA <- combined_df$MVPA + combined_df$LPA

# Rename the dataset

combined_dfternary <- combined_df

# Load required libraries

library(ggplot2)

library(ggtern)

# Define custom color gradient

custom_colors <- colorRampPalette(c("red", "orange", "yellow", "green", "blue"))(100)

# Generate the ternary plot with custom color gradient

ggtern(data = combined_dfternary, aes(x = PA, y = Sedentary, z = sleep, color = percentile_total1)) +

geom_point() + # Add points

geom_text(aes(label = round(percentile_total1, 1)), size = 3, hjust = 0.5, vjust = -1) + # Add labels, rounded to 1 decimal

theme_bw() + # Apply theme

labs(

title = "Ternary Plot of Movement Behaviors of 3 to 4-year-olds",# or Ternary Plot of Movement Behaviors of 1 to 2-year-olds

x ="Physical activity",

y = "Sedentary behaviour",

z = "Sleep",

color = "Percentile Total"

) +

scale_color_gradientn(colors = custom_colors) # Apply custom color gradient

## 3-dimensional isobar

# Load plotly

library(plotly)

# 3D scatter plot using MVPA, LPA, and Sedentary with color for percentile_total1

fig <- plot_ly(

data = combined_dfternary,

x = ~MVPA,

y = ~LPA,

z = ~Sedentary,

color = ~percentile_total1, # Use percentile_total1 for color

colors = colorRampPalette(c("red", "yellow", "green", "blue"))(100),

text = ~paste(

"MVPA:", round(MVPA, 2), "min/day", "<br>",

"LPA:", round(LPA, 2), "min/day", "<br>",

"SED:", round(Sedentary, 2), "min/day", "<br>",

"Sleep:", round(sleep, 2), "min/day", "<br>",

"Percentile Score:", round(percentile_total1, 2)

), # Show all variables in hover text

hoverinfo = "text", # Display only custom text in hover info

marker = list(size = 4)

) %>%

add_markers() %>%

layout(

title = "3D Isobars of 3 to 4-year-olds Data",

scene = list(

xaxis = list(title = "MVPA (min/day)"),

yaxis = list(title = "LPA (min/day)"),

zaxis = list(title = "SED (min/day)")

)

)

fig

## Shiny_app

#generate shiny_app

library(shiny)

# Load the dataset

combined_df <- read.csv("combined_df.csv")

# Define UI layout

ui <- fluidPage(

titlePanel("Movement Behaviour Combination for 3 to 4-year olds"),#OR Movement Behaviour Combination for 1 to 2-year olds

sidebarLayout(

sidebarPanel(

sliderInput("mvpa_input", label = "Moderate-to-vigorous physical activity (min/day):",

min = 50, max = max(combined_df$MVPA), value = 50, step = 5),#mvpa range based on your data

sliderInput("lpa_input", label = "Light physical activity (min/day):",

min = 270, max = max(combined_df$LPA), value = 270, step = 5),#lpa range based on your data

sliderInput("sedentary_input", label = "Sedentary time (min/day):",

min = 240, max = max(combined_df$Sedentary), value = 240, step = 5),#sedentary range based on your data

sliderInput("sleep_input", label = "Sleep duration (min/day):",

min = 520, max = max(combined_df$sleep), value = 520, step = 5),#sleep range based on your data

actionButton("submit", "Submit"),

textOutput("total_data_output")

),

mainPanel(

textOutput("percentile_output"),

sliderInput("percentile_input", label = "Percentile Value:",

min = min(combined_df$percentile_total1),

max = max(combined_df$percentile_total1),

value = median(combined_df$percentile_total1)),

# Modified instructions below

wellPanel(

style = "margin-top: 150px; margin-bottom: 20px; background-color: #f9f9f9; padding: 10px;",

HTML("

<strong>Instructions:</strong>

<ol>

<li>Adjust the sliders to the appropriate movement behaviour combination.</li>

<li>The Total of the four Movement Behaviours must be exactly <strong>1440 min/day</strong>.</li>

<li>Click <strong>Submit</strong> to generate the percentile score for the movement behaviour combination you have selected.</li>

</ol>

<strong>Notes:</strong>

<ul>

<li>A percentile score will not be generated if the Total of the four Movement Behaviours is not exactly <strong>1440 min/day</strong>.</li>

<li>A percentile score will not be generated for a movement behaviour combination that is not typically observed in 1 to 2-year-old children. For example, combinations that include high amounts of moderate-to-vigorous physical activity and low amounts of light physical activity.</li>

<li>The higher the percentile scores, the healthier the movement behaviour combination.</li>

</ul>

")

)

)

)

)

# Define server logic

server <- function(input, output, session) {

# Reactive to calculate total movement behaviors

total_data <- reactive({

input$mvpa_input + input$lpa_input + input$sedentary_input + input$sleep_input

})

# Display total MB

output$total_data_output <- renderText({

total <- total_data()

paste("Total of the four Movement Behaviours:", total)

})

# Display percentile or NA based on the input validity

output$percentile_output <- renderText({

total <- total_data()

if (total != 1440) {

return("Calculated Percentile: NA (Total minutes do not equal 1440)")

}

row_index <- which(combined_df$MVPA == input$mvpa_input &

combined_df$LPA == input$lpa_input &

combined_df$Sedentary == input$sedentary_input &

combined_df$sleep == input$sleep_input)

if (length(row_index) == 0) {

return("Calculated Percentile: NA (This combination does not exist)")

}

percentile_value <- combined_df$percentile_total1[row_index]

paste("Calculated Percentile:", percentile_value)

})

# Update the percentile slider based on the calculated percentile

observeEvent(input$submit, {

total <- total_data()

if (total != 1440) {

updateSliderInput(session, "percentile_input", value = NA)

return()

}

row_index <- which(combined_df$MVPA == input$mvpa_input &

combined_df$LPA == input$lpa_input &

combined_df$Sedentary == input$sedentary_input &

combined_df$sleep == input$sleep_input)

if (length(row_index) == 0) {

updateSliderInput(session, "percentile_input", value = NA)

return()

}

percentile_value <- combined_df$percentile_total1[row_index]

updateSliderInput(session, "percentile_input", value = percentile_value)

})

}

# Run the application

shinyApp(ui = ui, server = server)

# Ring-Fencing Approach vs. 3 Standard Deviation in Generating Predictive Grids

Using 3 to 4-year-olds data (n=1500), we compare the two methods for generating predictive grids: the ±3 standard deviation (3SD) approach used in Dumuid et al (2) and the percentile-based method (referred to as "ring-fencing" in the methods) used in Nkifarjam et al (1). As shown in the figure below, the 3SD approach created a grid that included large areas of “white space” where there were no empirical data points. This occurred because the method assumes a symmetric distribution around the mean, extending beyond the observed data into unrealistic or unobserved regions. In contrast, the ring-fencing approach created a more realistic and precise boundary, focusing on the actual data distribution and excluding extreme, unobserved combinations of movement behaviours. As illustrated in the figure, the ring fence approach follows the natural shape of the data more so than the 3SD approach.
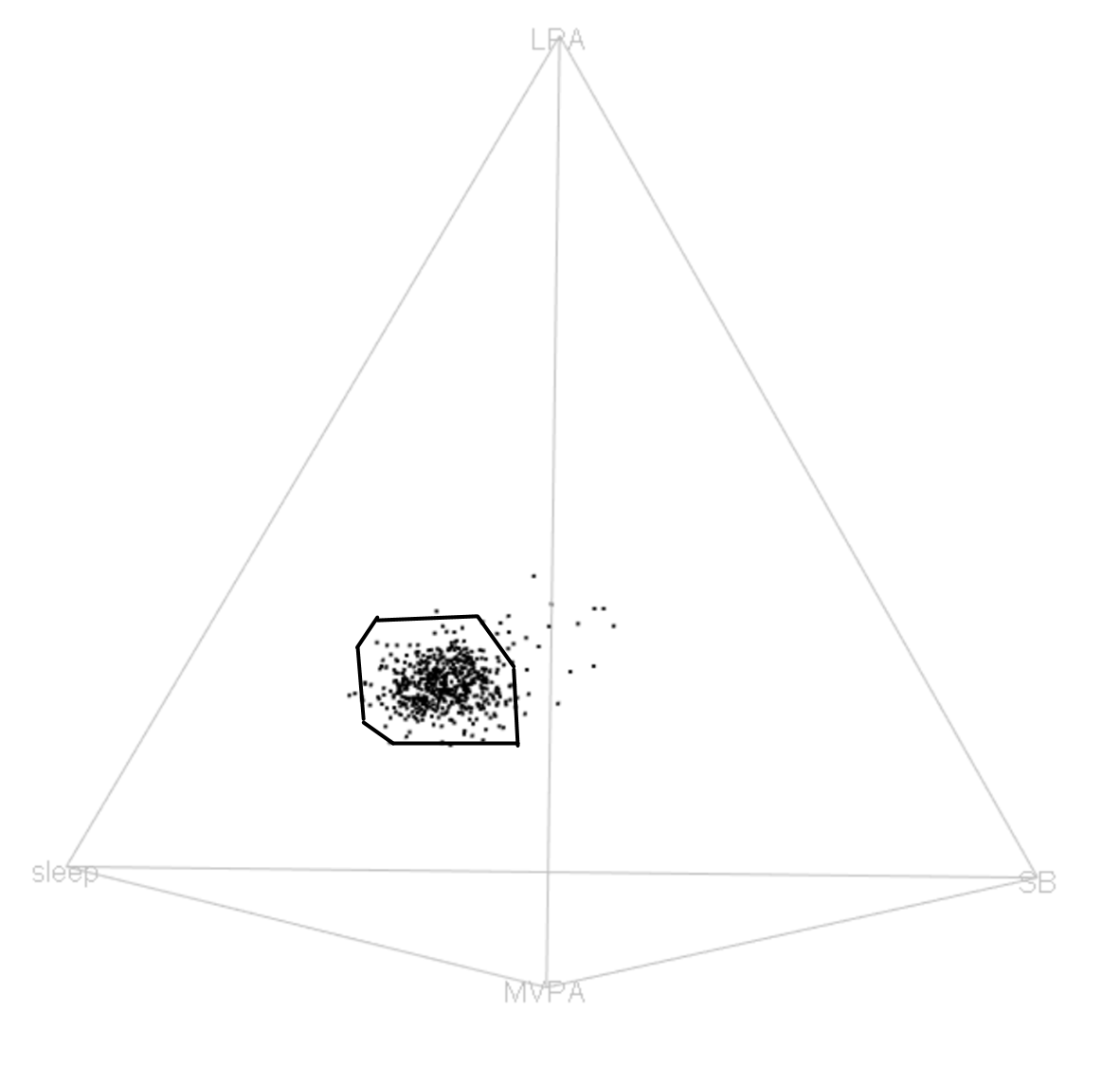

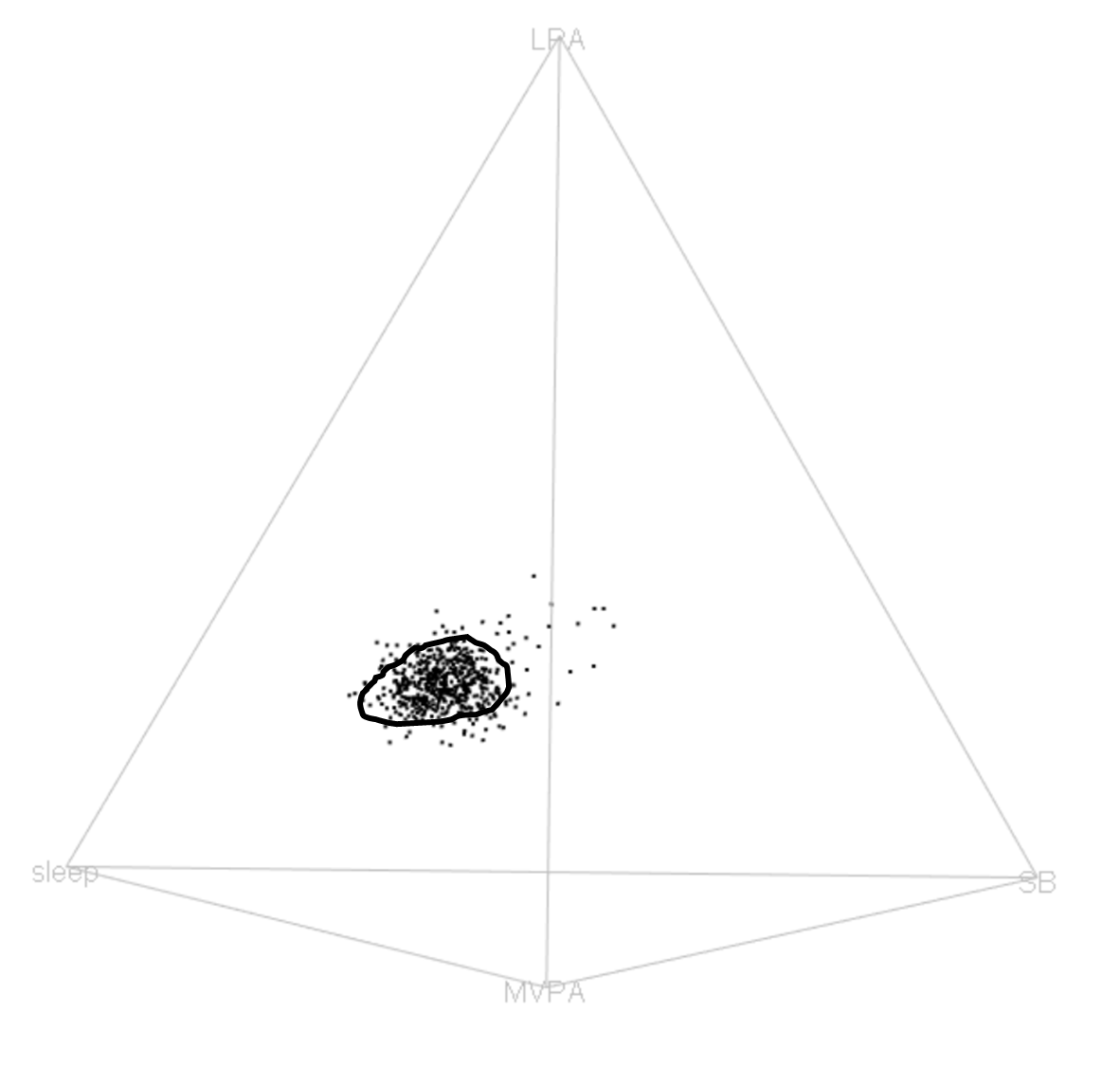


3 SD grid ring-fencing

*Figure Legend*: Original data for 1,500 participants in black and the predictive grid as a border

References

1. Nikfarjam A, Stanford T, Neumann A, Dumuid D, Neumann F, editors. Quality Diversity Approaches for Time-Use Optimisation to Improve Health Outcomes. Proceedings of the Genetic and Evolutionary Computation Conference; 2024.

2. Dumuid D, Olds T, Lange K, Edwards B, Lycett K, Burgner DP, et al. Goldilocks Days: optimising children’s time use for health and well-being. J Epidemiol Community Health. 2022;76(3):301-8.
