# Supplementary material for: Development and application of a scoring and visualization approach for 24-hour movement behaviours: an example based on social-emotional development in early years children
Source: Int J Behav Nutr Phys Act. 2026 Mar 24;23:46. doi: 10.1186/s12966-026-01907-y (PMC13137636; doi:10.1186/s12966-026-01907-y)
Supplement: Supplementary file 3 — Supplementary Material 3. [file 12966_2026_1907_MOESM3_ESM.doc]

STROBE Statement—Checklist of items that should be included in reports of ***cross-sectional studies***

|  | Item No | Recommendation | Page and line number |
| --- | --- | --- | --- |
| **Title and abstract** | 1 | (*a*) Indicate the study’s design with a commonly used term in the title or the abstract | The study’s design is mentioned in the abstract (Page 3, line 43). |
|  |  | (*b*) Provide in the abstract an informative and balanced summary of what was done and what was found | Abstract provides summary of objectives, methods, results, and conclusions (Page 3- 4). |
| Introduction | | |  |
| Background/rationale | 2 | Explain the scientific background and rationale for the investigation being reported | Background outlines the rationale for moving beyond binary guideline classifications (Page 5- 7, lines 73-140). |
| Objectives | 3 | State specific objectives, including any prespecified hypotheses | Objectives clearly stated at the end of the introduction, including purpose of developing a scoring system (Page 8, lines 141- 147). |
| Methods | | |  |
| Study design | 4 | Present key elements of study design early in the paper | Design described early in Methods as a cross-sectional study using SADEY data (Page 8, lines 150-153). |
| Setting | 5 | Describe the setting, locations, and relevant dates, including periods of recruitment, exposure, follow-up, and data collection | Settings, 7 countries, and cohorts (PATH-ABC, ACTNOW, Get-Up!, PLAYCE) described; data collection dates referenced (Page 8, lines 150-156). |
| Participants | 6 | (*a*) Give the eligibility criteria, and the sources and methods of selection of participants | (Page 9, lines 174-175). |
| Variables | 7 | Clearly define all outcomes, exposures, predictors, potential confounders, and effect modifiers. Give diagnostic criteria, if applicable | SDQ, movement behaviours, and covariates are defined clearly (Page 9, lines 166-172; page 10, lines 195-202, lines 206-210). |
| Data sources/ measurement | 8* | For each variable of interest, give sources of data and details of methods of assessment (measurement). Describe comparability of assessment methods if there is more than one group | Accelerometery, parent-reported sleep, and SDQ scoring are all described in Methods (Page 9, lines 167-170, lines 173-182; page 10, lines 196-200). |
| Bias | 9 | Describe any efforts to address potential sources of bias | Addressed via harmonization, imputation, and standardized protocols (Page 9, lines 175-178; page 9-10, lines 183-192; page 10, lines 206-208; and page 18, lines 372-382). |
| Study size | 10 | Explain how the study size was arrived at | Sample size reflects available cohort data; no formal power calculation provided (page 8, lines 150-154). |
| Quantitative variables | 11 | Explain how quantitative variables were handled in the analyses. If applicable, describe which groupings were chosen and why | ilr transformation and regression model handling explained (Page 11, lines 217-228). |
| Statistical methods | 12 | (*a*) Describe all statistical methods, including those used to control for confounding | Regression modelling and CoDA methods described (Page 11-12, lines 210-246). |
|  |  | (*b*) Describe any methods used to examine subgroups and interactions | Age-stratification; sex interactions tested and non-significant (page 9, lines 164). |
|  |  | (*c*) Explain how missing data were addressed | Missing cases excluded; flow diagram shown in supplementary Figure S1. |
|  |  | (*d*) If applicable, describe analytical methods taking account of sampling strategy | Not applicable (we did not perform any other additional analyses that don't reflect the main results). |
|  |  | (*e*) Describe any sensitivity analyses | None performed. |
| Results | | |  |
| Participants | 13* | (a) Report numbers of individuals at each stage of study—eg numbers potentially eligible, examined for eligibility, confirmed eligible, included in the study, completing follow-up, and analysed | The number of participants with missing data for each variable of interest is shown in the flow diagram in the supplemental file Figure S1). |
|  |  | (b) Give reasons for non-participation at each stage | Addressed in supplementary Figure S1. |
|  |  | (c) Consider use of a flow diagram | Supplementary Figure S1 provided. |
| Descriptive data | 14* | (a) Give characteristics of study participants (eg demographic, clinical, social) and information on exposures and potential confounders | Participant characteristics and confounders in Table 1 (Page 25). |
|  |  | (b) Indicate number of participants with missing data for each variable of interest | The number of participants with missing data for each variable of interest is shown in the flow diagram in the supplemental file Figure S1). |
| Outcome data | 15* | Report numbers of outcome events or summary measures | Outcome measures reported in Table 1 (Page 25). |
| Main results | 16 | (*a*) Give unadjusted estimates and, if applicable, confounder-adjusted estimates and their precision (eg, 95% confidence interval). Make clear which confounders were adjusted for and why they were included | Confounder-adjusted estimates and precision presented in Table 2 and Table S1; covariate selection explained (Pages 13–14, lines 272–302; Page 10, lines 206–208; Page 26, Table 2). |
|  |  | (*b*) Report category boundaries when continuous variables were categorized | Not applicable. |
|  |  | (*c*) If relevant, consider translating estimates of relative risk into absolute risk for a meaningful time period | Not applicable. |
| Other analyses | 17 | Report other analyses done—eg analyses of subgroups and interactions, and sensitivity analyses | No additional subgroup or sensitivity analyses. |
| Discussion | | |  |
| Key results | 18 | Summarise key results with reference to study objectives | Summary with reference to objectives (Page 15, lines 306–311). |
| Limitations | 19 | Discuss limitations of the study, taking into account sources of potential bias or imprecision. Discuss both direction and magnitude of any potential bias | (Page 17 and 18, lines 355 -393). Study limitation discussed. |
| Interpretation | 20 | Give a cautious overall interpretation of results considering objectives, limitations, multiplicity of analyses, results from similar studies, and other relevant evidence | (Page 15 and 16, lines 312-354). Interpretation of findings provided. |
| Generalisability | 21 | Discuss the generalisability (external validity) of the study results | (Page 17 and 18, lines 370-382). |
| Other information | | |  |
| Funding | 22 | Give the source of funding and the role of the funders for the present study and, if applicable, for the original study on which the present article is based | (Page 20, lines 432-447) Funding listed in Declarations. |

*Give information separately for exposed and unexposed groups.

**Note:** An Explanation and Elaboration article discusses each checklist item and gives methodological background and published examples of transparent reporting. The STROBE checklist is best used in conjunction with this article (freely available on the Web sites of PLoS Medicine at http://www.plosmedicine.org/, Annals of Internal Medicine at http://www.annals.org/, and Epidemiology at http://www.epidem.com/). Information on the STROBE Initiative is available at www.strobe-statement.org.
